# The value of Apolipoprotein B/Apolipoprotein A1 ratio for metabolic syndrome diagnosis in a Chinese population: a cross-sectional study

**DOI:** 10.1186/1476-511X-13-81

**Published:** 2014-05-14

**Authors:** Fangyuan Jing, Yingying Mao, Jing Guo, Zhenyu Zhang, Yingjun Li, Zhenhua Ye, Ye Ding, Jianbing Wang, Mingjuan Jin, Kun Chen

**Affiliations:** 1 Department of Epidemiology and Biostatistics, Zhejiang University School of Public Health, Hangzhou, Zhejiang, China; 2 Center for Disease Control and Prevention of Zhejiang Province, Hangzhou, Zhejiang, China

**Keywords:** Metabolic syndrome, Apolipoprotein ratio, ROC curve

## Abstract

**Background:**

The apoB/apoA1 ratio has been reported to be associated with the metabolic syndrome (MetS), and it may be a more convenient biomarker in MetS predicting. However, whether apoB/apoA1 ratio is a better indicator of metabolic syndrome than other biomarkers and what is the optimal cut-off value of apoB/apoA1 ratio as an indicator of metabolic syndrome in Chinese population remain unknown. Thus, we carried out the current study to assess the predictive value of apoB/apoA1 ratio and determine the optimal cut-off value of apoB/apoA1 ratio for diagnosing MetS in a Chinese population.

**Method:**

We selected 1,855 subjects with MetS and 6,265 individuals without MetS based on the inclusion and exclusion criteria from the China Health Nutrition Survey (CHNS) in 2009. MetS was identified based on the diagnostic criteria of International Diabetes Federation (2005). Logistic regression was used to estimate the association between the apoB/apoA1 ratio and risk of MetS, and receiver operating characteristics (ROC) curve analysis was performed to test the predictive value of apoB/apoA1 ratio and calculate the appropriate cut-off value.

**Results:**

Compared with the lowest quartile of apoB/apoA1 ratio, subjects in the fourth quartile had a higher risk of MetS in both men [odds ratio (OR) = 2.64, 95% confidence interval (CI) =1.82-3.83] and women (OR = 5.18, 95% CI = 3.87-6.92) after adjustment for potential confounders. The optimal cut-off value of apoB/apoA1 ratio for MetS detection was 0.85 in men and 0.80 in women. Comparisons of ROC curves indicated that apoB/apoA1 ratio was better than traditional biomarkers in predicting MetS.

**Conclusion:**

Our results suggest that, apoB/apoA1 ratio has a promising predictive effectiveness in detection of MetS. An apoB/apoA1 ratio higher than 0.85 in men and 0.80 in women may be a promising and convenient marker of MetS.

## Background

Metabolic syndrome (MetS) has become the fastest growing chronic disease worldwide [1-3], including China [4]. MetS is characterized as a cluster of risk factors for atherosclerosis including abdominal obesity, glucose intolerance, hypertension, hyper triglyceridaemia (TG) and low high-density lipoprotein cholesterol (HDL-C), all of which increase the risk of cardiovascular disease incidence and mortality [5]. Total cholesterol (TC), TG and HDL-C levels vary greatly with the dietary intake of fat, and moreover, measurements of TG and HDL-C need an at least 12-hours fast in clinical practice, which is not convenient for patients. Therefore, a more convenient biomarker with comparable diagnostic value for replacement is meaningful in clinic.

Apolipoprotein B (apoB) is present in atherogenic lipoproteins including very-low-density lipoprotein (VLDL), intermediate-density lipoprotein (IDL) and low density lipoprotein (LDL). Apolipoprotein A1 (apoA1) is a major constituent of HDL, an anti-atherogenic apolipoprotein [6,7]. Thus, the apoB/apoA1 ratio could represent the balance between atherogenic and anti-atherogenic lipoproteins. In the clinical setting, the apoB/apoA1 ratio can be measured at any time without fasting [8]. It implies that the apoB/apoA1 ratio may be a more convenient biomarker in MetS predicting.

Recently, several studies have reported that the apoB/apoA1 ratio is associated with metabolic syndrome in different ethnical groups [7-11]. However, only few studies with relative small sample size have evaluated the association between the apoB/apoA1 ratio and MetS in the Chinese population [9,10]. It remains unclear whether apoB/apoA1 ratio is a better indicator for identifying metabolic syndrome in the Chinese population, and there are no optimal cut-off values of apoB/apoA1 ratio for Chinese men and women yet.

To assess the association between apoB/apoA1 ratio and risk of MetS, compare the predictive effectiveness of apoB/apoA1 ratio with various lipid ratios in China and calculate the optimal cut-off values of apoB/apoA1 ratio for Chinese men and women, we analyzed the data from the China Health and Nutrition Survey 2009 (CHNS 2009) in current study.

## Results

### Demographic characteristics of participants with or without MetS

Demographic characteristics of the 1,855 participants with MetS and 6,265 without MetS are shown in Table 1. The prevalence of MetS in this study population was 22.8%. All demographic characteristics except gross household income were significantly different between the MetS and control groups (P <0.01).

**Table 1:** Demographic characteristics by subjects with and without MetS

	**metS- (n = 6265)**	**metS + (n = 1855)**	***p***
Age, mean (SD)	49.27(15.14)	55.72(12.68)	<0.0001
Male, n (%)	49.48	36.71	<0.0001
BMI^a^, mean (SD)	22.43(2.96)	26.47(3.16)	<0.0001
Urbanization index, %	66.61(19.40)	69.96(19.56)	<0.0001
Gross household income^b^, %			0.9161
Low	33.46	33.1	
Median	33.15	33.64	
High	33.39	33.28	
Education levels, %			<0.0001
Illiteracy	21.63	29.23	
Primary school	19.31	21.52	
Junior high school	34.04	29.45	
Senior high school and higher levels	25.03	19.8	
Smoke, %			<0.0001
Never	67.41	74.69	
Current	29.47	21.86	
Ever	3.11	3.45	
Drink, %			<0.0001
Never	65.98	72.44	
≤3 drink/month	12.26	8.83	
1-2 drink/week	7.86	6.5	
3-4 drink/week	4.08	3.74	
Almost every day	9.82	8.5	
Energy intake (kcal/day), mean (SD)	2204.13(1101.71)	2194.93(1330.65)	0.004
History of CVD, %	1.82	3.4	<0.0001
Diabetes, %	3.71	19.76	<0.0001
Hypertension, %	21.82	59.35	<0.0001

^a^body mass index = weight (kg)/height (m^2^).

^b^categorized by tertiles of gross household income at 19600.00 and 39799.12 yuan.

### Biomarkers and components of MetS across quartiles of apoB/apoA1 ratio

Table 2 presents the level of some clinical biomarkers for MetS across apoB/apoA1 ratio quartiles. Rising trend was observed in total cholesterol, uric acid, creatinine, ALT, hs-CRP, waist circumference, fasting glucose, HOMA-IR, systolic and diastolic blood pressure, LDL-C and triglyceride and all components except for HDL across the apoB/apoA1 ratio quartiles (P for trend < 0.001), while a negative relationship was found between apoB/apoA1 ratio quartiles and HDL (P for trend < 0.001).

**Table 2:** Biomarkers and components of MetS across quartiles of apoB/apoA1 ratio

	**Quartiles of apoB/apoA1 ratio**				
	**Q1 (<0.61)**	**Q2 (0.61-0.78)**	**Q3 (0.78-0.98)**	**Q4 (≥0.98)**	**P**_***trend***_^**a**^
n	2036	2026	2035	2023	
ApoB (g/l)	0.68 (0.19)	0.84 (0.18)	0.96 (0.19)	1.16 (0.23)	<0.001
ApoA1 (g/l)	1.40 (0.58)	1.20 (0.25)	1.09 (0.22)	0.95 (0.18)	<0.001
Total cholesterol(mmol/l)	4.26 (0.83)	4.63 (0.82)	4.96 (0.86)	5.55 (0.99)	<0.001
Uric acid (mg/dl)	282.96 (94.33)	293.50 (89.19)	314.10 (109.13)	334.63 (115.38)	<0.001
Urea (mmol/l)	5.34 (1.56)	5.42 (1.50)	5.48 (1.42)	5.47 (1.40)	0.225
Creatinine (umol/l)	83.48 (13.80)	85.07 (13.72)	86.55 (13.89)	88.93 (14.29)	<0.001
ALT (u/l)	19.00 (11.48)	20.21(11.91)	22.81 (14.17)	25.39 (15.63)	<0.001
hs-CRP	1.80 (5.08)	2.12 (6.42)	2.79 (12.55)	3.09 (5.37)	<0.001
Waist circumference (cm)	78.07 (9.51)	81.35 (9.83)	84.11 (9.81)	87.21 (10.02)	<0.001
Fasting glucose (mmol/l)	5.07 (1.01)	5.16 (1.06)	5.45 (1.55)	5.72 (1.81)	<0.001
HOMA-IR^b^	2.77 (4.16)	3.23 (5.56)	3.89 (7.41)	4.64 (8.71)	<0.001
Systolic BP (mm Hg)	120.39 (17.95)	123.42 (18.59)	125.51 (18.54)	129.15 (18.97)	<0.001
Diastolic BP (mm Hg)	78.02 (11.28)	79.96 (11.03)	81.12 (11.15)	83.01 (11.36)	<0.001
LDL-C (mmol/l)	2.27 (0.71)	2.81 (0.80)	3.11 (0.81)	3.70 (0.95)	<0.001
HDL-C(mmol/l)	1.69 (0.42)	1.50 (0.53)	1.33 (0.37)	1.22 (0.47)	<0.001
Triglyceride (mmol/l)	1.22 (1.25)	1.40 (1.20)	1.83 (1.57)	2.21 (1.64)	<0.001

^a^linear trend was adjusted by age and gender. P of age was adjusted by gender only. P of gender was adjusted for age only.

^b^HOMA-IR = fasting insulin(FINS)[mU/L] × fasting plasma glucose (FPG)[mmol/L])/22.5.

### Association between apoB/apoA1 ratio and risk of MetS

Table 3 summarizes the associations between apoB/apoA1 ratio and risk of MetS. We found a significant association between higher apoB/apoA1 ratio and increased risk of MetS. Compared with the lowest quartile, subjects in the fourth quartile had a 4.24 fold higher risk of MetS (Model 3). This significant association was present in both men and women, but was stronger among women (OR = 5.18, 95% CI = 3.87-6.92) than among men (OR = 2.64, 95% CI = 1.82-3.83). There was evidence of a statistically significant monotonic trend. (*P*_trend_ values <0.001).

**Table 3:** Adjusted ORs (95% CI) for the associations between apoB/apoA1 ratio and risk of MetS

	**Quartiles of apoB/apoA1 ratio**				
**Models**	**Q1 (<0.61)**	**Q2 (0.61-0.78)**	**Q3 (0.78-0.98)**	**Q4 (≥0.98)**	**P**_***trend***_
All subjects					
Model 1^a^	Reference	1.40 (1.11-1.76)	2.57 (2.07-3.19)	4.30 (3.48-5.32)	<0.0001
Model 2^b^	Reference	1.37 (1.08-1.73)	2.59 (2.08-3.23)	4.28 (3.44-5.31)	<0.0001
Model 3^c^	Reference	1.35 (1.06-1.73)	2.69 (2.13-3.89)	4.24 (3.37-5.32)	<0.0001
Men					
Model 1^a^	Reference	1.04 (0.69-1.57)	1.96 (1.36-2.84)	2.92 (2.05-4.16)	<0.0001
Model 2^b^	Reference	0.98 (0.64-1.48)	1.97 (1.36-2.87)	2.89 (2.01-4.13)	<0.0001
Model 3^c^	Reference	0.94 (0.61-1.45)	1.93 (1.31-2.85)	2.64 (1.82-3.83)	<0.0001
Women					
Model 1^a^	Reference	1.58 (1.19-2.09)	2.74 (2.10-3.58)	4.95 (3.78-6.47)	<0.0001
Model 2^b^	Reference	1.58 (1.19-2.11)	2.79 (2.12-3.66)	4.93 (3.75-6.48)	<0.0001
Model 3^c^	Reference	1.58 (1.17-2.14)	2.99 (2.24-4.00)	5.18 (3.87-6.92)	<0.0001

^a^Model 1 was adjusted by age, gender and BMI.

^b^based on Model 1, Model 2 was additionally adjusted by smoking, drinking, urbanization index, educational levels, dietary energy intake everyday, gross household income per year.

^c^based on Model 2, Model 3 was additionally adjusted by history of CVD, hypertension, diabetes.

### Diagnostic performances of apoB/apoA1 ratio

**Figure 1:**
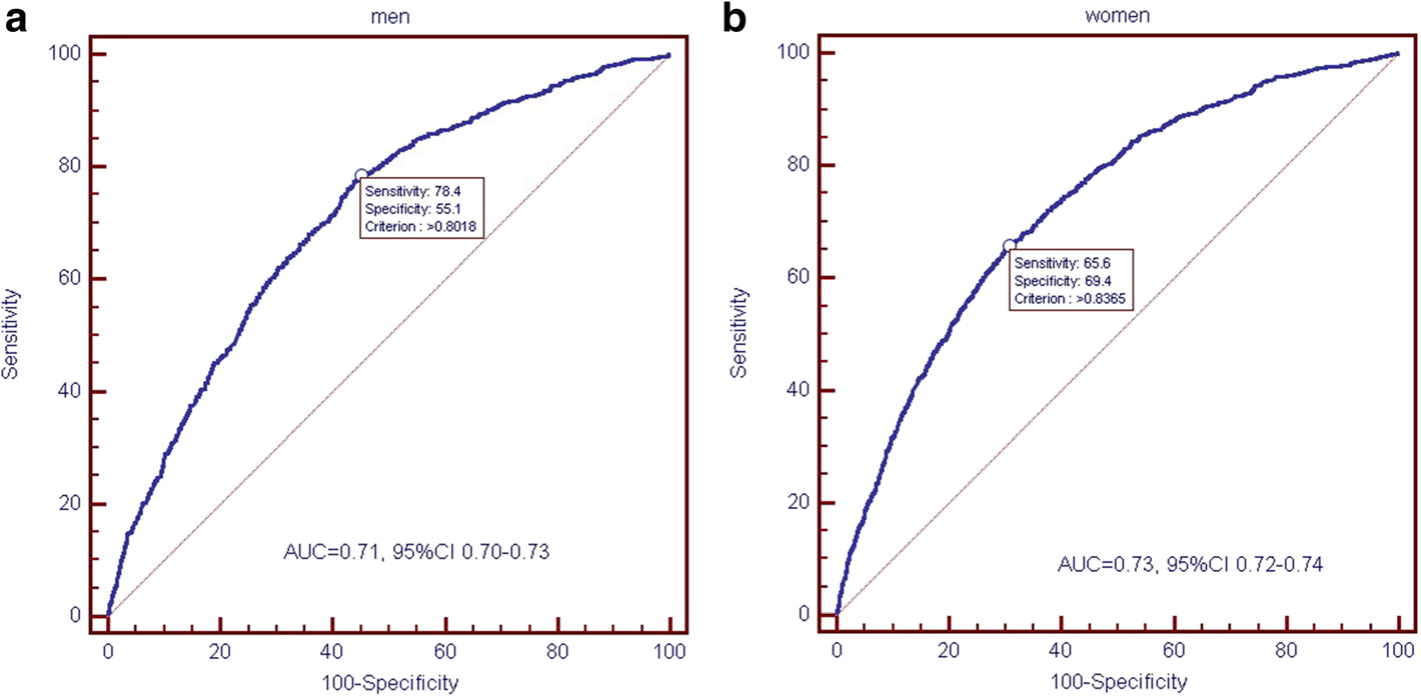
Receiver operating characteristic curve of apoB/A1 ratio for metabolic syndrome (MetS) in men (a) and women (b).

ROC analysis was performed to assess the diagnostic value of apoB/apoA1 ratio for MetS. Overall, the optimal cut-off value of apoB/apoA1 ratio for MetS detection was 0.82, with a sensitivity of 71.0% and a specificity of 61.9% (AUC = 0.72, 95% CI 0.71-0.73). The optimal apoB/apoA1 ratio cut-off values among men and women for detecting MetS were 0.80 (AUC = 0.71, 95% CI 0.70-0.73) and 0.84 (AUC = 0.73, 95% CI 0.72-0.74), respectively. The sensitivities were 78.4% and 65.6%, the specificities were 55.1%, 69.4%, respectively (Figure 1). After adjustment for potential confounders, the ORs in participants with an apoB/apoA1 ratio >0.82 overall, >0.80 in men, and > 0.84 in women were 2.71 (95% CI = 2.37-3.10), 2.18(95% CI = 1.76-2.71), and 2.73 (95% CI = 2.31-3.24), respectively. Most of the ORs remained significant when stratified by age, glucose status, history of CVD and obesity status (Table 4). Similarly, most of the ORs for components of MetS were also significant after adjusted for the covariates mentioned above.

**Table 4:** ORs (95% CI) for MetS and individual components based on the optimal cut-off value of ApoB/apoA1 ratio

	**All subjects**		**Men**		**Women**	
	**OR (95% CI)**	**P**	**OR (95% CI)**	**P**	**OR (95% CI)**	**P**
**OR**^**a **^**for MetS**						
Overall	2.71 (2.37-3.10)	<0.001	2.18 (1.76-2.71)	<0.001	2.73 (2.31-3.24)	<0.001
Age category						
<50	2.65 (2.12-3.31)	<0.001	2.52 (1.80-3.53)	<0.001	2.75 (2.05-3.68)	<0.001
≥50	2.62 (2.21-3.09)	<0.001	1.95 (1.47-2.60)	<0.001	2.68 (2.17-3.30)	<0.001
Glucose status						
NGT	3.27 (2.73-3.92)	<0.001	2.59 (1.90-3.54)	<0.001	3.01 (2.42-3.75)	<0.001
IFG	1.68 (1.27-2.23)	<0.001	1.35 (0.87-2.11)	0.182	1.92 (1.32-2.80)	<0.001
Diabetes	1.66 (1.05-2.63)	0.03	1.31 (0.71-2.40)	0.386	3.89 (1.16-4.90)	0.018
History of CVD						
No	2.70 (2.36-3.09)	<0.001	2.13 (1.71-2.66)	<0.001	2.74 (2.31-3.26)	<0.001
Yes	3.21 (1.52-7.25)	<0.001	5.41 (1.44-20.35)	0.012	2.50 (0.80-7.79)	0.114
Obesity status^b^						
BMI < 24	2.68 (2.11-3.39)	<0.001	1.54 (0.95-2.50)	0.082	3.30 (2.50-4.36)	<0.001
BMI ≥ 24	2.50 (2.13-2.93)	<0.001	2.22 (1.74-2.82)	<0.001	2.33 (1.88-2.88)	<0.001
**OR**^**a **^**for components**^**c**^						
Waist	1.33 (1.17-1.51)	<0.001	1.29 (1.06-1.57)	0.011	1.24 (1.04-1.46)	0.015
Glucose	1.52 (1.36-1.70)	<0.001	1.52 (1.29-1.79)	<0.001	1.53 (1.31-1.79)	<0.001
BP	1.21 (1.09-1.35)	<0.001	1.15 (0.99-1.34)	0.077	1.12 (0.96-1.29)	0.153
TG	2.82 (2.54-3.13)	<0.001	2.41 (2.07-2.81)	<0.001	2.93 (2.53-3.40)	<0.001
HDL-C	4.01 (3.57-4.51)	<0.001	3.90 (3.20-4.75)	<0.001	3.92 (3.39-4.53)	<0.001

^a^all ORs were adjusted for age, gender, BMI, smoking, drinking, urbanization index, educational levels, dietary energy intake everyday, gross household income per year.

^b^Chinese criterion.

^c^all events are defined according to IDF 2005 criteria (see Methods for details).

Furthermore, to check whether the apoB/apoA1 ratio was better than other biomarkers for MetS detection, we also compared AUC of their ROC curves (Table 5). The AUC of apoB/apoA1 ratio was significantly higher than other lipid biomarkers (P < 0.001).

**Table 5:** Diagnostic performances of different indicators

	**All subjects**		**Men**		**Women**	
	**AUC**^**a**^	**P**^**b**^	**AUC**^**a**^	**P**^**b**^	**AUC**^**a**^	**P**^**b**^
ApoB/apoA1	**0.72 (0.71-0.73)**	Standard	**0.71 (0.70-0.73)**	Standard	**0.73 (0.72-0.74)**	Standard
ApoB	0.68 (0.67-0.69)	<0.001	0.66 (0.64-0.68)	<0.001	0.69 (0.68-0.70)	<0.001
ApoA1	0.61 (0.59-0.62)	<0.001	0.62 (0.61-0.64)	<0.001	0.61 (0.59-0.62)	<0.001
LDL	0.56 (0.55-0.58)	<0.001	0.54 (0.52-0.55)	<0.001	0.58 (0.56-0.59)	<0.001
HDL/LDL	0.70 (0.69-0.71)	<0.001	0.68 (0.66-0.69)	<0.001	0.72 (0.71-0.74)	0.569

^a^area under curve.

^b^p value for the comparison with AUC of ApoB/apoA1, and modified by the method of Sidak.

## Discussion

In this study, we estimated the association between apoB/apoA1 ratio and metabolic syndrome in a relatively large Chinese population, compared the predictive effectiveness of apoB/apoA1 ratio with various traditional lipid ratios, and calculated the optimal cut-off value of apoB/apoA1 ratio in a Chinese population.

We found a significant association between higher apoB/apoA1 ratio and risk of MetS. Compared with the lowest quartile, subjects in the fourth quartile ratio had a higher risk of MetS with an OR of 4.24 (95%CI = 3.37-5.32). Apolipoproteins are structural and functional proteins in the lipoprotein particles. ApoB and apoA1, main constituents of atherogenic and anti-atherogenic lipoproteins, play important roles in cholesterol and lipid transportation [6,7]. A number of prospective studies have shown that high apoB/apoA1 ratio may be a promising marker for predicting the occurrence of future cardiovascular events, such as myocardial infarction and stroke [11,12]. In addition, ApoB concentration and apoB/apoA1 ratio were found to be associated with risk of MetS and its components and were independent of conventional risk factors in several previous studies [7,9,13-15]. Our results were consistent with these findings.

Few studies have focused on the appropriate cut-off values for MetS diagnosis in these study populations. Pitsavos C et al. [16] suggested a ratio of 0.73 as an optimal cut-off for predicting MetS, with a sensitivity of 74% and a specificity of 67% in a Greek population. Chang et al. [17] reported the sex-specific optimal apoB/apoA1 ratio cut-off values in their study, 0.65 in men and 0.62 in women. In addition, an apoB/apoA1 ratio of more than 0.7 in men and 0.6 in women was indicated by Walldius [11] as a signal of subsequent occurrence of myocardial infarction. In the current study, optimal cut-off values calculated for diagnosing metabolic syndrome were 0.82 in all subjects, 0.85 in men, and 0.80 in women, respectively. In consistence with previous studies, these cut-off values remained their diagnostic utility in most situations after stratification by potential confounding factors such as age and obesity status. In our study, however, the cut-off values are likely to be higher than those reported in previous studies. One possible explanation of the discrepancy is that our study had a higher median of apoB/apoA1 ratio compared with other studies, with evidence of a higher apoB level and a lower apoA1 level in the current population (Table 2). Furthermore, it has been reported that prevalence of MetS in Asians is higher than that in Caucasians after adjustment for body size [18]. Studies showed accumulation of intra-abdominal fat is easier among Asian people [13,14], which may also implied a higher average lipid level in serum among Asians people including Chinese.

The comparisons of ROC curves suggested that apoB/apoA1 ratio, as a marker of MetS, was better than other traditional biomarkers. To our knowledge, only one large cross-sectional study has compared the diagnostic values of different lipid ratios for MetS prediction in Korea, suggesting that non-HDL-C/HDL ratio might be a better predictor, which was also confirmed in our study. However, TC, TG and HDL-C levels vary greatly with the dietary intake of fat, and the measurements of TG and HDL-C need an at least 12-hour fast in clinical practice which is inconvenient for patients in clinic. Therefore, the non-HDL-C/HDL ratio calculated according to TC and HDL levels is also not convenient in clinic. In contrast, measurements of apoB and apoA1 do not require fasting samples and their measurement methods are internationally standardized in reference materials traceable to the World Health Organization. Therefore, apoB/apoA1 ratio could still be an easily accessible tool instead of TG or HDL.

Several limitations of this study deserve mention. First, the causal relationship between apoB/apoA1 ratio and risk of MetS cannot be conclusively determined due to the cross-sectional design. Second, we lacked information about previous treatment on HDL-C or triglyceride, which might influence the diagnosis of MetS. Finally, owing to the design of CHNS, we could not validate the predictive value of apoB/apoA1 ratio in another independent population. Despite of these limitations, this is the first study to compare the predictive effectiveness of apoB/apoA1 ratio with various traditional lipid ratios and suggest optimal cut-off value of apoB/apoA1 ratio in identifying subjects with MetS in China. Our sample size was large for detecting associations between apoB/apoA1 ratio and risk of MetS.

In conclusion, the present study provides the first evaluation of optimal cut-off values of apoB/apoA1 ratio in identifying MetS patients in Chinese population. We found that apoB/apoA1 ratio was associated with risk of MetS and observed a better predictive effectiveness of apoB/apoA1 ratio compared with other traditional lipid biomarkers, perhaps reflecting a promising and convenient biomarker for diagnosing MetS. However, additional studies are needed to confirm these findings.

## Method

### Study population

Subjects were selected from the CHNS 2009. Details of the CHNS 2009 have been described elsewhere [15,19,20]. In brief, CHNS, an ongoing large-scaled and household-based survey, started in 1989 and followed up every 2–4 years [19]. A stratified multistage, random cluster method was used as a sampling strategy in nine provinces that vary substantially in geography, economic development, public resources (including Heilongjiang, Liaoning, Shandong, Jiangsu, Henan, Hubei, Hunan, Guizhou and Guangxi), covering nearly 56% of the whole Chinese population.

A total of 9,511 records of fasting blood information from different participants were obtained in the dataset of CHNS2009. The exclusion criteria for selecting subjects were: (i) age < 18 years old (n = 845); (ii) pregnant women (n = 62); (iii) apoB/apoA1 ratio >3 (n = 10); abnormal kidney (serum creatinine >130 mmol/l in men or >120 mmol/l in women), abnormal liver function (ALT, TP, ALB ≥2.5*upper limit of normal value); (vi) individuals who had Thyromegaly (n = 9), cancer (n = 5), urinary system disease (n = 21). In addition, we also excluded the subjects whose diagnostic information for MetS was missing. Finally, 1,855 participants with MetS and 6,265 individuals without MetS were included in our study.

The study was approved by institutional review board from the University of North Carolina at Chapel Hill, the National Institute of Nutrition and Food Safety, China-Japan Friendship Hospital, the Chinese Center for Disease Control and Prevention, and Ministry of Health. Every participant provided a written informed consent.

### Definition of metabolic syndrome

MetS was defined according to the diagnostic criteria of International Diabetes Federation in 2005. Participants were diagnosed as MetS patients when they suffered from central obesity (waist circumference ≥90 cm in men and ≥80 cm in women), and met two or more of the following criteria: (i) triglyceride level ≥150 mg/dl (1.7 mmol/L) or specific treatment for its abnormality; (ii) HDL-C level <40 mg/dl (1.03 mmol/L) in men or <50 mg/dl (1.29 mmol/L) in women or specific treatment for its abnormality; (iii) systolic blood pressure (SBP) ≥130 mmHg and/or diastolic blood pressure (DBP) ≥85 mmHg or treatment of previously diagnosed hypertension and (iv) fasting glucose level ≥100 mg/dl (5.6 mmol/L) or previously diagnosed type 2 diabetes. Additional file 1: Figure S1 shows the details of subject selection.

### Measurement for variables used in analysis

Height and weight were measured when subjects were wearing light indoor clothing without shoes. Body mass index (BMI) was calculated as weight in kilograms divided by the square of height in meters. Waist circumference (WC) was measured at a level midway between the costal margin and the iliac crest at the end of a normal expiration. Blood pressure was measured in the right arm with the use of a mercury sphygmomanometer after the participant had been sitting quietly for five minutes. Urbanization index was categorized according to its quartiles and calculated from 12 components, covering social, cultural, economic and community-level physical environments across time and place [19]. Gross household income was grouped as low, median and high levels based on the tertiles. Educational background was classified as illiteracy, primary school, junior high school, senior high school and higher levels. Smoking status was categorized as never, current and ever. Alcohol drinking was defined as follows: never, ≤3 drink/month, 1–2 drink/week, 3–4 drink/week and almost everyday.

For each participant, an overnight fasting blood sample was drawn by venipuncture. Serum glucose was tested immediately in the local hospitals Serum glucose, Triglycerides (TG), high-density lipoprotein cholesterol (HDL-C) apoB and apoA1 were measured by GPO-PAP, enzymatic methods, and Immunoturbidimetric methods (Hitachi 7600 automated analyzer, Kyowa, Japan), respectively. Details of laboratory analysis were reported in “China Health and Nutrition Survey (CHNS), Manual for Specimen Collection and Processing” (http://www.cpc.unc.edu/projects/china/data/datasets/Blood%20Collection%20Protocol_English.pdf) and “A list of biomarkers and methods used to measure them” (http://www.cpc.unc.edu/projects/china/data/datasets/Biomarker_Methods.pdf).

### Statistical analysis

Continuous variables were expressed as mean ± SD, and categorical variables were calculated as proportion (%). Student’s *t*-test and Chi-square test were used to compare the continuous and categorical demographic characteristics between subjects with and without MetS, respectively. All participants were categorized into four groups according to the quartiles of apoB/apoA1 ratio in the controls. Biochemical characteristics of the population across the apoB/apoA1 quartiles were compared using one-way analysis of variance (ANOVA). Linear regression was used to assess the association between the apoB/apoA1 ratio and other biomarkers in serum. We used unconditional logistic regression models to estimate the odds ratios (ORs) and 95% confidence intervals (CIs). We used three models to estimate the association between apoB/apoA1 ratio and risk of MetS, and different confounding factors were included for adjustment in these three models, respectively. In addition, receiver operating characteristics (ROC) curve was performed to calculate the area under the curve (AUC), evaluating the diagnostic value of apoB/apoA1 ratio. Optimal cut-off values of apoB/apoA1 ratio were determined by Youden index [21] (sensitivity + specificity - 1). Furthermore, we conducted a ROC comparison analysis to compare the utility of apoB/apoA1 ratio with other biomarkers for identifying MetS. The AUC and optimal points were determined using MedCalc version 11.4.2.0 for Windows (MedCalc Software, Mariakerke, Belgium). All data analyses except ROC analysis were conducted using STATA version 11.0 (STATA Corp, College Station, Texas) and SAS version 9.2 (SAS Institute Inc., Cary, NC). All tests were two sided and *P* < 0.05 was considered statistically significant.

## Competing interests

The authors declare that they have no competing interests.

## Authors’ contributions

JFY and MYY were the main investigator of this study and draft the manuscript and prepared the final version of the manuscript. CK conceived the study and took part in study design. GJ took part in study design and statistical analysis. ZZY and LYJ helped with data collection and interpretation of the data. YZH and DY helped to draft the manuscript. JMJ and WJB have helped in the revision of the manuscript. All authors read and approved the final manuscript.

## Supplementary Material

Additional file 1: Figure S1 Flow chart of sample selection. Click here for file
