# Supplementary material for: The value of Apolipoprotein B/Apolipoprotein A1 ratio for metabolic syndrome diagnosis in a Chinese population: a cross-sectional study
Source: Lipids Health Dis. 2014 May 14;13:81. doi: 10.1186/1476-511X-13-81 (PMC4041140; doi:10.1186/1476-511X-13-81)
Supplement: Additional file 1: Figure S1 — Flow chart of sample selection. [file 1476-511X-13-81-S1.doc]

IDF2005

**CHNS2009**

**（n=9511）**

Exclusion criteria:

-age<18 years (n=845)

-pregnant (n=62)

-Thyromegaly (n=9)

-abnormal creatinine, ALT, TP, ALB (n=265) ^a^

-cancer (n=5)

-urinary system disease (n=21)

-apoB/apoA-1 ratio>3 (n=10)

**Subjects excluded (n=1217)**

**Potential subjects for current study**

**(n=8294)**

Incomplete data for MetS diagnosis (n=174)

**Eligible subjects with MetS**

**(n=1855)**

**Eligible subjects without MetS**

**(n=6265)**

^a^. abnormal creatinine (serum creatinine >130mmol/l in men or >120mmol/l in women), abnormal liver (ALT, TP, ALB >2.5*upper limit of normal value)

**Figure1. Flow chart of sample selection**
